# Relationships Between Fungal and Plant Communities Differ Between Desert and Grassland in a Typical Dryland Region of Northwest China

**DOI:** 10.3389/fmicb.2018.02327

**Published:** 2018-10-02

**Authors:** Jianming Wang, Chen Chen, Ziqi Ye, Jingwen Li, Yiming Feng, Qi Lu

**Affiliations:** ^1^College of Forestry, Beijing Forestry University, Beijing, China; ^2^Institute of Desertification Studies, Chinese Academy of Forestry, Beijing, China

**Keywords:** plant communities, soil fungal communities, functional groups, desert, grassland

## Abstract

The relationships between soil fungal and plant communities in the dryland have been well documented, yet the associated difference in relationships between soil fungal and plant communities among different habitats remains unclear. Here, we explored the relationships between plant and fungal functional communities, and the dominant factors of these fungal communities in the desert and grassland. Soil fungal functional communities were assessed based on fungal ITS sequence data which were obtained from our previous study. The results showed that the total, saprotrophic and pathotrophic fungal richness were predominantly determined by plant species richness and/or soil nutrients in the desert, but by MAP or soil CN in the grassland. AM fungal richness was only significantly related to soil nutrients in two habitats. The total and saprotrophic fungal species compositions were mainly shaped by abiotic and spatial factors in the desert, but by plant and abiotic factors in the grassland. Pathotrophic fungal species composition was more strongly correlated with plant and spatial factors in the desert, but with spatial and abiotic factors in the grassland. AM fungal species composition was more strongly correlated with MAP in the grassland, but with no factors in the desert. These results provide robust evidence that the relationships between soil fungal and plant communities, and the drivers of soil fungal communities differ between the desert and grassland. Furthermore, we highlight that the linkages between soil fungal and plant communities, and the drivers of soil fungal communities may also be affected by fungal traits (e.g., functional groups).

## Introduction

As one of the most fundamental components of biomes, the interactions between aboveground and belowground communities play crucial roles in regulating terrestrial biodiversity and ecosystem functions (van der Heijden et al., 2006; Van Dam and Heil, 2011; Wagg et al., 2014). There has been increasing evidences which strongly support the direct and indirect interactions between soil fungal and plant assemblages (Tedersoo et al., 2014; Chen et al., 2017; Yang et al., 2017). It is widely reported that plant communities influence soil fungal communities by generating diverse exudates, litter, microhabitats or host specificity (Hooper et al., 2000; Dickie, 2007; Millard and Singh, 2010; Eisenhauer et al., 2011). By contrast, soil fungal communities can indirectly regulate plant diversity through altering soil nutrient availability (van der Putten et al., 2013), or even directly promote plant diversity by mediating plant coexistence (Bever et al., 2015; Bennett and Cahill, 2016). Therefore, understanding the internal linkages between soil fungal and plant communities is essential to predict the response of the ecosystem to global environmental changes. Despite a great number of studies, to date the internal linkages between fungal and plant communities remain elusive.

It is believed that plant communities are mainly determined by climatic and historical factors (Wang et al., 2009; Fernandez-Going et al., 2013; Sundqvist et al., 2013). By contrast, soil fungal communities may be influenced by a variety of environmental factors in addition to plant factors (Tedersoo et al., 2014; Barberan et al., 2015; Chen et al., 2017), such as soil (Liu et al., 2015; Ma et al., 2017), climatic (Maestre et al., 2015; Newsham et al., 2016) and historical factors (Chen et al., 2017; Wang J.M. et al., 2017). Moreover, the relative importance of these factors to soil fungal communities vary across geographical scales and habitat types (Prober et al., 2015; Yang et al., 2017). Hence, the relationships between plant and fungal diversity may vary geographically. For example, plant diversity strongly influence fungal diversity in the grassland of northern China (Chen et al., 2017), whereas it has no significant effect on fungal diversity across grasslands worldwide (Prober et al., 2015). Habitat differentiation would also affect the dispersal ability of hosting microbes (Cermeno and Falkowski, 2009), and the influence of environmental gradients may cause the species composition and drivers of microbial communities to differ markedly among habitat types (Gao et al., 2017; Wang X.B. et al., 2017). Furthermore, the fungal traits (such as functional types, dispersal ability, identity, etc.) may affect the relationships between plant and fungal communities (Peay et al., 2010, 2013; Tedersoo et al., 2014; Yang et al., 2017). Therefore, taking fungal traits such as functional group types into account might provide new insights into the microbial assembly mechanism. Taken together, the relationships between soil fungal (especially different functional fungi) and plant communities are also likely to vary across and within habitat types.

The northern Xinjiang of China is one of the world’s largest dryland regions, which is almost entirely covered by desert and grassland. As a consequence of the global environmental changes, these ecosystems are expanding and simultaneously suffering extreme weather events frequently (Easterling et al., 2000; Dai, 2013), which may lead to substantial changes on soil fungal community assembly structures (Maestre et al., 2015). Indeed, we found that plant communities differ significantly between the grassland and desert. For instance, the main constructive plant species in the grassland are herbaceous plants (e.g., *Stipa capillata*, *Stipa breviflora*), while plant communities in the desert are predominantly dominated by semi-arbor and xeric shrub plants, such as *Haloxylon ammodendron* or *Calligonum mongolicum*, etc. Systematic differences in climatic and edaphic conditions between the desert and grassland may also induce the change of soil fungal communities in these habitats (Chen et al., 2017; Wang J.M. et al., 2017). These differences in soil, plant and climate may change the linkages between soil fungal and plant communities. However, few studies to date have focused on elucidating the associated difference in the relationships between soil fungal and plant communities across these habitats in Northwest China.

To explore the linkages between soil fungal and plant communities in the grassland and desert, we selected 32 of desert sites and 30 of grassland sites from a typical dryland region in Northwest China. Soil fungal functional communities were assessed based on fungal ITS sequence data which were obtained from our previous study (Wang J.M. et al., 2017). In this study, we mainly attempt to address the following three questions: (1) Do the species richness and composition of soil fungal functional groups differ between the desert and grassland? (2) Do the relationships between soil fungal (especially different functional fungi) and plant communities differ between the desert and grassland? (3) What are the drivers of soil fungal functional communities in the desert and grassland, respectively?

## Materials and Methods

### Study Region

The northern Xinjiang of China, one of the world’s largest dryland ecosystems, covers more than 450,000 km^2^. The climate is controlled by the continental air mass, changing from arid and semi-arid to semi-humid zones, and from warm temperate and temperate to plateau temperate zones. Together, the main climate can be characterized as arid and semi-arid, with high variability of precipitation and temperature due to the highly rugged and dissected topography. Therefore, the vegetation types in study region are mainly desert and grassland, whereas other vegetation types only cover a relatively small area.

### Site Selection and Field Sampling

**FIGURE 1:**
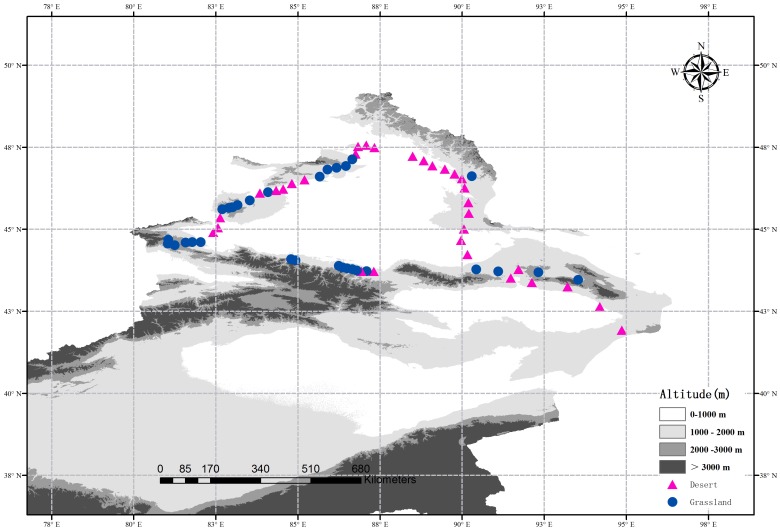
Location of the desert (red triangles) and grasslands (blue dots) sampling sites across a typical dryland region in Northwest China. The altitude data is provided by Data Center for Resources and Environmental Sciences, Chinese Academy of Sciences (RESDC) (http://www.resdc.cn) and then we created the maps using ArcGIS 10 (http://www.esri.com/software/arcgis).

Indeed, the methods of site selection, field sampling and the determinations of soil attributes have been described in the previous study (Wang J.M. et al., 2017). In 2016, Wang J.M. et al. (2017) selected 62 sites from a typical dryland region in northern Xinjiang, China, and obtained soil fungal sequences and soil attributes data of these sites. In this study, we divided these 62 sites into two subsets: 32 sites in the desert and 30 sites in the grassland (**Figure 1**). At each site, five 1 m × 1 m quadrats within an area of 10 m × 10 m were randomly established from the representative vegetation. Meanwhile, the altitude and geographic factors (latitude and longitude) were recorded, and the plant coverage was calculated visually for each quadrat. All vascular plant species and their individuals were measured and recorded at each quadrat, and then summarized at the site level. Finally, three soil samples (10 cm in depth) were randomly collected from each quadrat, and then the 15 soil samples were mixed together into a composite sample. All composite samples were sieved through a 2 mm mesh and then separated into two portions: one portion was stored in thermal insulated boxes (at 4°C) for determining the soil physicochemical properties, and the other was stored at -20°C until DNA extraction.

We selected the mean annual temperature (MAT) and mean annual precipitation (MAP) as climatic variables. Both the data of MAP and MAT were extracted from WorldClim global climate database using the geographic coordinates of each site^1^ (with a resolution of 1 km × 1 km). Soil attributes (soil pH (pH), total phosphorus and nitrogen (TSN and TSP), total organic carbon (TOC); soil available nitrogen (AN), moisture content (SM), soil N: P (NP) and C: N (CN) ratios) have been measured by Wang J.M. et al. (2017). Information on the soil, plant and climate of desert and grassland is summarized in **Supplementary Table S1**.

### Molecular and Bioinformatics Analysis

Both the fungal raw and clean sequence data have been obtained by Wang J.M. et al. (2017). Briefly, the fungal genomic DNA was extracted from the well-mixed fresh soil samples (0.5 g) using E.Z.N.A. soil DNA kits (OMEGA, United States) following the manufacturer’s instructions. We amplified the fungal internal transcribed spacer (ITS) region using the primer set ITS1F (5′-CTTGGTCATTTAGAGGAAGTAA-3′) and ITS2 (5′-TGCGTTCTTCATCGATGC-3′). These primers contained a set of 8-nucleotide barcode sequences unique to each sample. The PCR program was as follows: 95°C for 5 min, 25 cycles at 95°C for 30 s, 55°C for 30 s, and 72°C for 30 s with a final extension of 72°C for 10 min. The amplicon mixture was applied to the MiSeq Genome Sequencer (Illumina, San Diego, CA, United States). Amplicons were extracted from 2% agarose gels and purified using the AxyPrep DNA Gel Extraction Kit (Axygen Biosciences, Union City, CA, United States) according to the manufacturer’s instructions and quantified using QuantiFluor^TM^ -ST (Promega, United States). Purified amplicons were pooled in equimolar and paired-end sequenced (2 × 300) on an Illumina Miseq PE300 sequencing platform (Illumina, Inc., CA, United States) according to the manufacturer’s recommendations.

In this study, all the fungal high-quality sequence data were reprocessed within the QIIME package, with the procedure described by Caporaso et al. (2012) and Miao et al. (2016). Briefly, the sequences were clustered into operational taxonomic units (OTUs) based on the threshold of 97% identity using UCLUST. Chimeric sequences were identified and removed using Usearch (Version 9.2). The taxonomy of each ITS gene sequence was analyzed by comparison against sequences within the UNITE database (Version 7.0) using UCLUST. Wang J.M. et al. (2017) reported that the fungal high-quality sequence ranged from 19,581 to 45,083 per sample, with an average of 37, 914 reads per sample. To eliminate the influence for the different read numbers among samples, we randomly selected subsets of the 19,581 sequences in this study to identify the Fungal OTUs again. Then the major functional groups (i.e., pathotrophic, saprotrophic, arbuscular mycorrhizal (AM) and ectomycorrhizal (EM) fungi) were determined following the criteria of Tedersoo et al. (2014) and Nguyen et al. (2016a). As EM fungi occurred very rarely, only AM fungal data were included in this study. Finally, Wang J.M. et al. (2017) have submitted these associated fungal DNA sequences to SRA of NCBI database under accession number SRP119964.

### Statistical Analysis

The differences in the richness and relative abundance of the dominant genera (with relative abundance > 1.0%) and major functional groups between the desert and grassland were estimated by one-way analysis of variance (One-way ANOVA). We further exhibited the variation in the relative abundance of the dominant genera (with relative abundance > 1.0%) and major functional groups between the desert and grassland within the “ggplot2 package”. Next we used principal coordinates of neighbor matrices (PCNM) to obtain the spatial variables (Dray et al., 2006). Then the pairwise Bray–Curtis distance for the plant and soil fungal functional communities (Hellinger-transformed the abundance data) and environmental Euclidean distance were calculated within “vegan” package (Oksanen et al., 2016). Furthermore, we estimated the pairwise geographic distance within the fossil package according to the GPS coordinates.

The linear and quadratic regressions were constructed to explore the relationships between the fungal functional richness, plant (richness and coverage), and abiotic variables (soil and climate). Then the Akaike information criterion (AIC) values were used to determine the more fitted model (with a 10 unit smaller AIC value; Burnham and Anderson, 2002). The stepwise multiple regressions were used to further evaluate the relative effects of plant and abiotic factors. The quadratic terms of the explanatory variables would be included in the initial models to account for quadratic relationships. To prevent data overfitting, all variables were subjected to forward-selection until *P* < 0.05, and any variables with an inflation factor greater than 10 were removed to avoid the strong collinearity among variables (Quinn and Keough, 2002). When more than one variable was retained in the final model, the independent contribution of each retained variable would be assessed through hierarchical partitioning analysis.

Meanwhile, we tested the differences in the species compositions of fungal functional groups between the desert and grassland using Permutational analysis of variance (10000 permutations; Clarke and Ainsworth, 1993) within the “vegan” package. Mantel correlation analysis was used to explore the relationship between community composition and individual environmental variable. Then the multiple regressions on matrices (MRM) approach was conducted to further examine the relations of community composition to the plant, abiotic and geographic distance within “ecodist” package (Goslee and Urban, 2007; Lichstein, 2007). To prevent data overfitting, all variables were subjected to forward-selection until *P* < 0.05. When more than one variable was retained in the final model, the independent contribution of each retained variable would be assessed through hierarchical partitioning analysis.

Finally, the vectors of the plant and fungal community composition were derived from the principal coordinate analysis (PCoA) based on the Bray–Curtis dissimilarity matrix. To reduce dimensionality, only the first 15 (10 in desert and grassland) of vectors of the plant community composition were used in the following analysis which represented more than 70% of the total variation. Then we conducted variation partitioning analysis based on distance-based redundancy analysis (dbRDA) to estimate the relative contribution of the plant (species richness, coverage, PCoA vectors), abiotic factors (soil and climate) and spatial variables (positive PCNM vectors) to the species composition (PCoA vectors) of fungal functional groups within “vegan” package. In addition, all variables were subjected to forward-selection until *P* < 0.05 using the “packfor” package before variation partitioning analysis (Dray et al., 2009).

## Results

A total of 1,211,922 fungal high-quality sequences were identified from 62 samples, which were grouped into 5,584 fungal OTUs. Of these 5,584 fungal OTUs, 1820 OTUs (691,266 sequence) were identified as 8 functional (trophic) groups, which occupied more than 50% of the total fungal sequences (**Supplementary Table S2** and **Supplementary Figure S1**). Furthermore, saprotrophic (1,010 OTUs), pathotrophic (179 OTUs) and arbuscular mycorrhizal fungi (189 OTUs) were the most dominant functional groups, which accounted for 63.4% of the total functional sequences (**Supplementary Table S2** and **Supplementary Figure S1**). Additionally, a total of 413 fungal genera were identified from all soil samples. Of these 413 fungal genera, the 12 most dominant genera (with relative abundance > 1.0%) were Fusarium, Preussia, Alternaria, Mortierella and so on (**Supplementary Figure S2**).

### Species Richness and Compositions of Soil Fungal Functional Groups in the Desert and Grassland

Of these 5,584 fungal OTUS, 3,755 (625,688 sequences) were recovered from desert samples and 4,617(586,234 sequences) were recovered from grassland samples (**Supplementary Table S2**). Furthermore, the fungal OTUs per desert sample changed from 313 to 836 (with an average of 530.22 ± 21.03 OTUs), while the fungal OTUs per grassland sample changed from 275 to 888 (with an average of 657.43 ± 26.71 OTUs). We found that the relative abundance of several dominant genera such as Fusarium, Mortierella, and Aspergillus showed significant group differences between the desert and grassland (*P* < 0.05).

**FIGURE 2:**
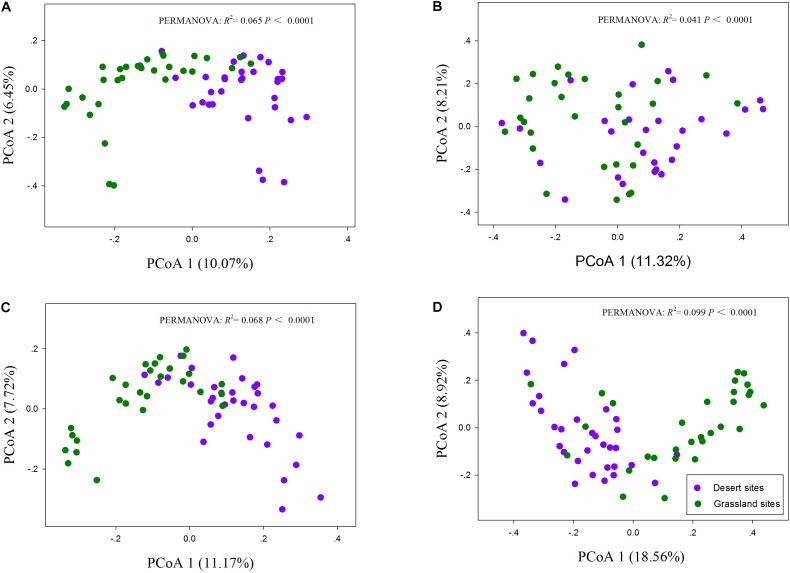
The principal coordinate analysis (PCoA) of total **(A)**, AM **(B)**, saprotrophic **(C)**, and pathotrophic **(D)** fungal communities.

The One-way ANOVA confirmed that the species richness of the total and AM fungi were significantly lower in the desert than in the grassland (*P* < 0.05; **Table 1**), whereas saprotrophic and pathotrophic fungal richness did not differ significantly between the desert and grassland (*P* > 0.05). Moreover, the relative abundance of AM fungi also showed significant group differences between the desert and grassland (*P* < 0.05, **Supplementary Figure S3**). Two-dimensional plot of PCoA showed that the patterns of all fungal functional communities varied distinctly between the desert and grassland (**Figure 2**). The Permutational analysis of variance (PERMANOVA) further demonstrated that the species compositions of the total (*R*^2^ = 0.065, *P* < 0.001), AM (*R*^2^ = 0.041, *P* < 0.001), saprotrophic (*R*^2^ = 0.068, *P* < 0.001), and pathotrophic (*R*^2^ = 0.099, *P* < 0.001) fungi differed significantly between the desert and grassland.

**Table 1:** Summary statistics of the richness of total fungi and major functional groups in the desert, grassland and whole region.

Fungal richness	Desert	Grassland	*F*	*P*
Total fungi	530.22 ± 21.03	657.43 ± 26.71	7.13	<0.001
Arbuscular mycorrhiza (AM)	7.46 ± 1.51	26.90 ± 3.05	16.21	<0.0001
Saprotroph	137.38 ± 5.66	157.40 ± 6.58	2.71	>0.05
Pathotroph	24.38 ± 1.32	27.33 ± 1.16	1.38	>0.05


### Influence of Plant, Soil and Climatic Factors on the Species Richness of Soil Fungal Functional Groups in the Desert and Grassland

**FIGURE 3:**
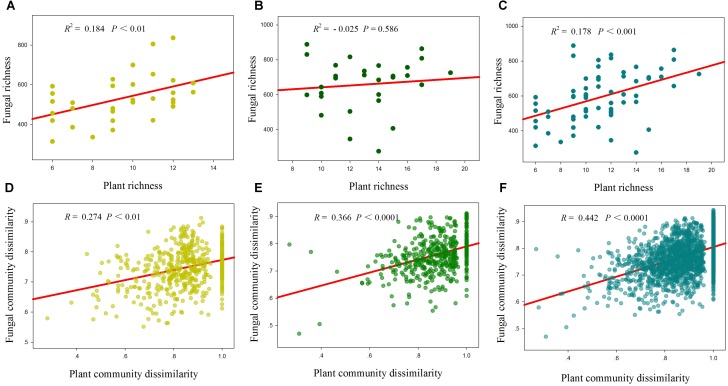
Relationships between plant species richness and fungal richness **(A–C)**, and between plant community dissimilarity and fungal community dissimilarity **(D–F)** in the desert **(A,D)**, grassland **(B,E)** and whole region **(C,F)**.

Total, saprotrophic and pathotrophic fungal species richness were significantly linearly increased with plant species richness in the desert and whole region (*P* < 0.05, **Supplementary Table S3** and **Figure 3**), whereas all had no significant correlations with plant species richness in the grassland (*P* > 0.05, **Supplementary Table S3** and **Figure 3**). AM fungal richness had no significant correlations with plant species richness in the desert and grassland (*P* > 0.05, **Supplementary Table S3**). The correlations of these fungal diversities with environmental factors also differed between the desert and grassland (**Supplementary Table S4**). Stepwise multiple regressions revealed that total fungal richness was significantly predicted by plant species richness in the desert, by MAP in the grassland, and by TSN, TOC^2^ and plant species richness in the whole region (**Table 2**). Saprotrophic and pathotrophic fungal richness were significantly predicted by CN and plant species richness in the desert, by MAP in the grassland, and by CN, MAP^2^ and plant species richness in the whole region (**Table 2**). AM fungal richness were prominently predicted by CN in the grassland, by TSN in the desert, and by NP in the whole region (**Table 2**).

**Table 2:** Stepwise multiple regressions of the species richness of different functional fungi with environmental variables in the desert, grassland and the whole region.

	Trophic group	Variable retained in the model and its individual contribution (%)	Model *R*^2^_adj_	Model *P*
Whole	Total fungi	TSN (13.8), TOC^2^ (12.2), Plant species richness (16.1)	0.391	<0.0001
	Arbuscular mycorrhiza^2^	NP	0.276	<0.001
	Saprotroph	CN (11.8), MAP^2^ (11.9), Plant species richness (22.2)	0.431	<0.0001
	Pathotroph	CN (18.1), MAP^2^ (4.9), Plant species richness (18.2)	0.382	<0.0001
				
Desert	Total fungi	Plant species richness	0.184	<0.01
	Arbuscular mycorrhiza^2^	TSN	0.129	<0.05
	Saprotroph	CN (13.8), Plant species richness (19)	0.283	<0.01
	Pathotroph	CN (22.1), Plant species richness (20.8)	0.391	<0.001
				
Grassland	Total fungi	MAP	0.263	<0.001
	Arbuscular mycorrhiza^1^	CN	0.107	<0.05
	Saprotroph	MAP	0.286	<0.01
	Pathotroph	MAP	0.111	<0.05


### Influence of Plant, Abiotic and Spatial Factors on Species Compositions of Soil Fungal Functional Groups in the Desert and Grassland

Mantel correlation analysis showed that total, saprotrophic and pathotrophic fungal species compositions were significantly related to plant species composition in the desert, grassland and the whole region (**Supplementary Table S3** and **Figure 3**). AM fungal community composition was significantly correlated with plant community composition in the grassland and the whole region, but not in the desert (**Supplementary Table S3**). Furthermore, almost all fungal functional community compositions (in addition to AM fungi in the desert) were significantly related to some selected environmental variables (i.e., MAP, MAT, and TSN, among others; **Supplementary Table S5**). Of explanatory variables, these fungal community compositions were more strongly correlated with geographic distance in the desert, whereas with abiotic factors in the grassland and whole region (**Table 3** and **Supplementary Table S5**).

**Table 3:** Results of the multiple regressions on distance matrices (MRM) for species compositions of different functional fungi in the desert, grassland and whole region.

	Trophic groups	Variable retained in the model and its individual contribution (%)	Model *R*^2^	Model *P*
Whole	Total fungi	Geographic distance (8.6), Plant dissimilarity (7.8),	0.585	<0.0001
		Plant coverage (7.9), CN (8.4), pH (6.3), MAP (19.5)		
	Arbuscular mycorrhiza	Geographic distance	0.042	<0.0001
	Saprotroph	Geographic distance (6.8), Plant dissimilarity (4.8),	0.491	<0.0001
		Plant coverage (7.5), CN (8.4), pH (5.8), MAP (15.8)		
	Pathotroph	Geographic distance (3.6), Plant dissimilarity (9.5),	0.425	<0.0001
		Plant species richness (4.6), SM (10), MAP (14.9)		
				
Desert	Total fungi	Geographic distance (16.2), TSN (3.4), CN (18.1), pH (6.2), MAT (7.1)	0.509	<0.0001
	Arbuscular mycorrhiza	No variable retained in the final model		
	Saprotroph	Geographic distance (10.4), CN (14.9), pH (5.3), MAT (8.3)	0.387	<0.0001
	Pathotroph	Geographic distance (6.6), Plant dissimilarity (8.5)	0.151	<0.0001
				
Grassland	Total fungi	Plant dissimilarity (8.5), Plant coverage (6.5), MAP (27.7)	0.427	<0.0001
	Arbuscular mycorrhiza	MAP	0.119	<0.001
	Saprotroph	Plant coverage (6.5), MAP (30.5)	0.369	<0.0001
	Pathotroph	Geographic distance (6.7), AN (10.8)	0.175	<0.0001


*MAP, mean annual precipitation; MAT, mean annual temperature; SM, soil moisture content; TSN, soil total nitrogen; TOC, soil total organic carbon; AN, soil available nitrogen; CN, soil carbon: nitrogen ratio. Geographic distance was log-transformed*.

The final multiple regressions on matrices analysis (MRM) showed that the all fungal functional species compositions (excluding AM fungi) were significantly explained by various combinations of geographic distance, plant and abiotic factors (**Table 3**). The total fungal community composition was significantly predicted by geographic distance and abiotic factors in the desert, whereas by plant and abiotic factors in the grassland (**Table 3**). AM fungal composition was significantly explained by MAP in the grassland, but by no factors in the desert (**Table 3**). Both geographic distance and abiotic variables were retained into the final MRM model for saprotrophic fungal communities in the desert, whereas geographic distance and plant factors were included into the model in the grassland (**Table 3**). Furthermore, the pathotrophic community composition was significantly predicted by geographic distance and plant factors in the desert, by geographic distance and AN in the grassland (**Table 3**). Variation partitioning analysis further demonstrated that plant, spatial and abiotic factors together explained 6.4–23.9%, 9.9–26.2%, 12.6–27.9% of the variations in species compositions of total, AM, saprotrophic and pathotrophic fungal communities in the desert, grassland and whole region, respectively (**Supplementary Figure S4**).

## Discussion

### Species Richness and Compositions of Soil Fungal Functional Groups Differ Between the Desert and Grassland

There has been increasing evidences that the soil fungal diversity and composition vary across habitat types (Chen et al., 2017; Gao et al., 2017; Ma et al., 2017). In our study, we found that species compositions of all fungal functional groups significantly differed between the desert and grassland. Furthermore, we also observed that the total and AM fungal richness were drastically lower in the desert than in the grassland. Together, we confirm that soil fungal functional communities vary across habitat types in the dryland of Northwest China. Notably, saprotrophic and pathotrophic species compositions were significantly different between the desert and grassland, whereas these fungal diversities did not differ significantly between the two habitats. Given that the variation in species composition among sites may result from species replacement and richness difference (Legendre, 2014), these results may suggest that different saprotrophic and pathotrophic species (at the same richness level) inhabit different habitats (Gao et al., 2017). From all above, we propose that the distribution patterns of soil microbial species may also be influenced by microbial traits (e.g., functional groups) in dryland regions.

Alternatively, we observed the systematic differences in soil, plant and climatic conditions between the desert and grassland. Therefore, this imply that the reasons for differences in the species richness and compositions of fungal functional groups are as follows: first, the difference in plant factors would result in the variation in plant hosts or organic substrates types, which in turn can induce the changes of the species diversity and compositions of soil fungal functional group (Wardle, 2006; Eisenhauer et al., 2011). Second, the difference in climatic and soil conditions between the two habitats may directly result in the shifts in these fungal communities (Matsuoka et al., 2016; Xu et al., 2016), and it also can lead to substantial changes of these fungal communities through affecting plant communities.

### Relationships Between the Soil Fungal Functional and Plant Communities Differ Between the Desert and Grassland

The coupled or uncoupled relationships between plant and fungal biodiversity have been observed by many previous studies (Vogelsang et al., 2006; Schnitzer et al., 2011; Peay et al., 2013; Tedersoo et al., 2014; Chen et al., 2017). In agreement with Chen et al. (2017), we observed that total, saprotrophic and pathotrophic fungal richness were significantly related to plant species richness in the whole region. This suggests that even relatively small individuals of dryland plants would be able to provide complementary underground niches to support higher saprotrophic and pathotrophic species richness through generating diverse exudates, litter and microhabitats (Dickie, 2007; Millard and Singh, 2010; Waring et al., 2015). We also found that total, saprotrophic and pathotrophic fungal richness were positively correlated with plant species richness in the desert, but not in the grassland. In addition, plant species richness was retained into the final model of these functional fungi in the desert. This suggests that plant species richness may be a key limited factor in controlling these fungal diversities in the desert, but not in the grassland.

It has been reported that plant communities play important roles for regulating fungal communities in the grassland at larger regional (Chen et al., 2017) and global scales (Prober et al., 2015). In our study, the species compositions of all functional fungi had significant correlation with plant species composition in the whole region, as reported in previous studies (Chen et al., 2017; Gao et al., 2017). MRM further revealed that plant species composition was retained into all final models (except for AM fungi), supporting the resource diversity and niche differentiation hypothesis which may be applicable to these fungal communities (Wardle et al., 2004; Nguyen et al., 2016b). We observed that AM fungal species composition had a significant response to plant species composition in the grassland, but not in the desert. Plant factors were included into the final model for saprotrophic fungi in the grassland, but not in the desert. By contrast, plant species composition was only retained into the final model of pathotrophic fungi in the desert. This may imply that the linkages between plant and these fungal compositions differ between the two habitats.

Some studies have reported that the fungal and plant biodiversity may be largely uncoupled (Wardle, 2006), and the inconsistent findings among different studies may result from the difference in spatial scales of surveys (Prober et al., 2015). However, the subsets of data in the desert and grassland covered similar spatial scales in our study, which demonstrate that the habitat differentiation could also induce the relationships between plant and these fungal communities to differ across different studies. Taken together, we provide robust evidence that the relationships between plant and these fungal communities vary across habitat types in the dryland regions.

It should also be noted that all plant factors were excluded from the final models for AM fungal species richness and composition in the desert, grassland and the whole region. In addition, plant factors also play different roles in shaping other fungal functional communities. For example, plant richness was the most important factor in shaping saprotrophic and pathotrophic fungal richness in the whole region, but not for AM fungal richness. This supports the viewpoints that the linkages between plant and fungal communities may also be affected by fungal traits (e.g., functional groups).

### Driving Factors of Soil Fungal Functional Communities Differ Between the Desert and Grassland

It is widely reported that the species richness and compositions of different functional fungi were influenced by extensive ranges of environmental variables (Rousk et al., 2010; Tedersoo et al., 2014; He et al., 2016; Wang J.M. et al., 2017), whereas the relative importance of these factors may vary across geographic regions and scales. In the whole region, species richness of all functional fungi were determined by various combinations of plant and abiotic factors, which confirmed previous reports. However, the major driving factors of these fungal diversities varied between the desert and grassland. For instance, the total, saprotrophic and pathotrophic fungal richness were mainly explained by plant species richness and abiotic factors in the desert, but only by abiotic factors in the grassland. From the result that the soil nutrient, MAP and plant species richness were obviously higher in the grassland than in the desert, we propose that the difference in these fungal diversities between the two habitats may result from above resource limitations (Tilman, 1982).

Multiple regressions on matrices showed that the community compositions of all functional fungi were mainly determined by plant, abiotic and spatial factors in the whole region, consistent with previous studies (Pellissier et al., 2014; Yang et al., 2017). However, the driving factors related to these fungal species compositions also differed between the desert and grassland. For example, geographic distance was retained into the final model for all models (except for AM fungi) in the desert, but only into the model for pathotrophic fungi in the grassland. Given that the influence of spatial distance may reflect the effect of historical processes (Martiny et al., 2006), these findings may indicate that historical processes (i.e., spatial isolation, dispersal limitation) play different roles in shaping soil fungal community structures between the desert and grassland.

Of environmental factors, climatic factors have been considered as major driving factors of microbial communities in global drylands (Maestre et al., 2015), while some studies found that soil factors had dominant influence on fungal communities in other regions (Liu et al., 2015; Ma et al., 2017). Total and saprotrophic fungal community structures were more strongly related to soil factors in the desert. However, these fungal community structures were more strongly related to MAP in the grassland, which consistent with the findings of Shi et al. (2014) and Timling et al. (2014). Although water availability has been considered as the key limited factor in dryland regions, we found MAP could only determine the fungal communities in the grassland. Perhaps it was because of the extremely high evapotranspiration and gravel coverage in the desert that the majority of MAP became non-available precipitation. We further found that the soil CN range (1.25 ∼ 34.17) in the desert was three times wider than in the grassland (5.29 ∼ 16.12). Conversely, the MAP range (43 ∼ 226) was 98.9% lower in the desert than in the grassland (94 ∼ 458). From these results, we propose that the resource niche gradient length may be responsible for the differences in these fungal community structures in the dryland (Gao et al., 2017). Taken together, we highlight that the driving factors of fungal functional communities significantly differ between the desert and grassland.

Variation partitioning analysis showed that abiotic and spatial factors explained more variation in the species compositions of different functional fungi in all habitats. These results are inconsistent with the findings in larger-scale grassland (Chen et al., 2017), but they support other reports (Prober et al., 2015; Tedersoo et al., 2016; Yang et al., 2017). We suggest that these obviously conflicting findings may also result from the differences in environmental gradient length in addition to spatial scales. From all above, we confirm that these fungal compositions were more strongly driven by abiotic and spatial factors. A significant proportion of variation in these fungal compositions can be explained by plant factors, indicating that plant factors also play non-negligible roles in influencing such fungal community structures in the dryland of China.

Finally, we also observed that the drivers of species richness and composition vary among fungal functional groups, even within a single habitat. Soil, plant and climate play different roles in shaping these fungal communities. Thus, we suggest the dominant driving factors of soil fungal communities may vary remarkably depending on fungal traits (e.g., functional group) in dryland regions.

## Conclusion

Our study attempts to explore the difference in relationships between plant and soil fungal functional communities between the desert and grassland by applying the consistent inquiry scale and analytical methods. Species richness of all functional fungi were significantly related to plant species richness in the desert, but not in the grassland. Plant species richness was a key limited factor in controlling these fungal diversities in the desert, but not in the grassland. This suggests that the linkages between plants and fungi differ between the desert and grassland. Furthermore, the total, saprotrophic and pathotrophic fungal species compositions were controlled by various combinations of plant, abiotic and spatial factors in the desert and grassland. Taken together, we highlight that the drivers of soil fungal functional communities differ between the desert and grassland. Finally, we also emphasize that the linkages between soil fungal and plant communities, and the drivers of soil fungal communities may also be influenced by fungal traits (e.g., functional groups) and environmental gradient length.

## Author Contributions

JW and JL designed the study. JW, JL, and QL developed the methods. JW and ZY performed the field investigation and collected the data. JW and YF conducted the analyses. JW and CC wrote the paper.

## Conflict of Interest Statement

The authors declare that the research was conducted in the absence of any commercial or financial relationships that could be construed as a potential conflict of interest.

## Footnotes

1 http://www.worldclim.org
