# Supplementary figures and images for: Relationships Between Fungal and Plant Communities Differ Between Desert and Grassland in a Typical Dryland Region of Northwest China
Source: Front Microbiol. 2018 Oct 2;9:2327. doi: 10.3389/fmicb.2018.02327 (PMC6176009; doi:10.3389/fmicb.2018.02327)

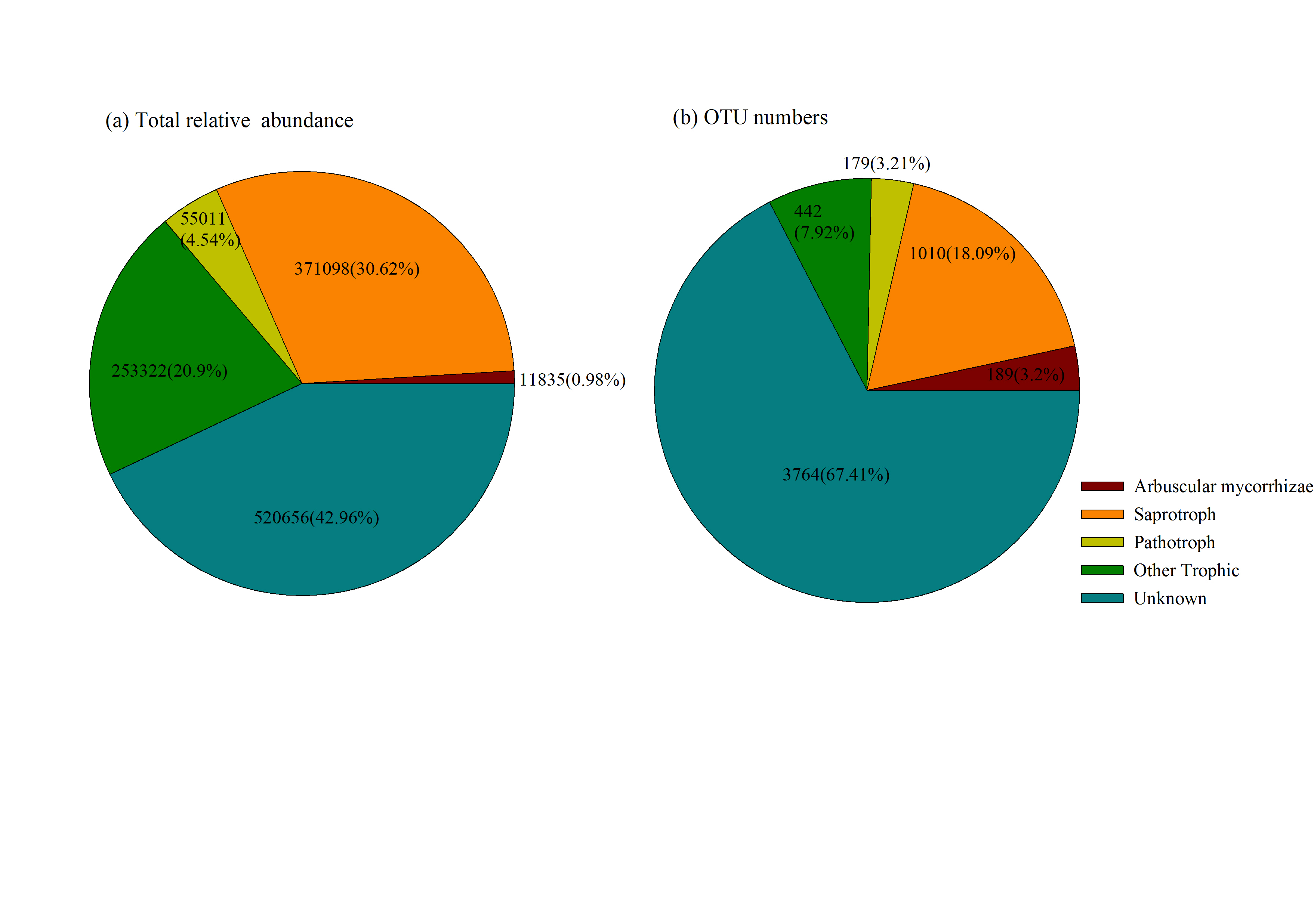

Supplement: Supplementary file 1 [file Image_1.JPEG]

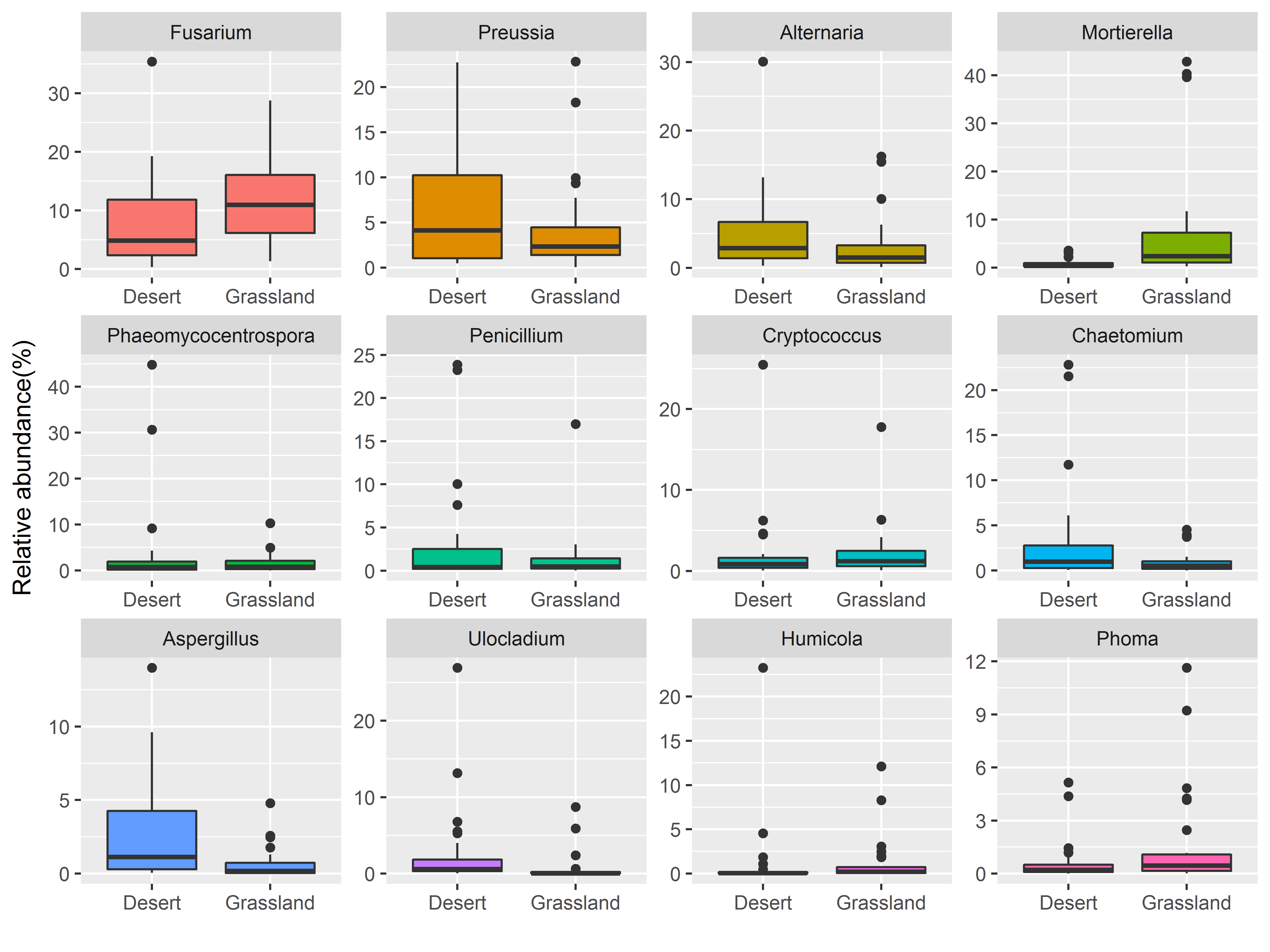

Supplement: Supplementary file 2 [file Image_2.TIFF]

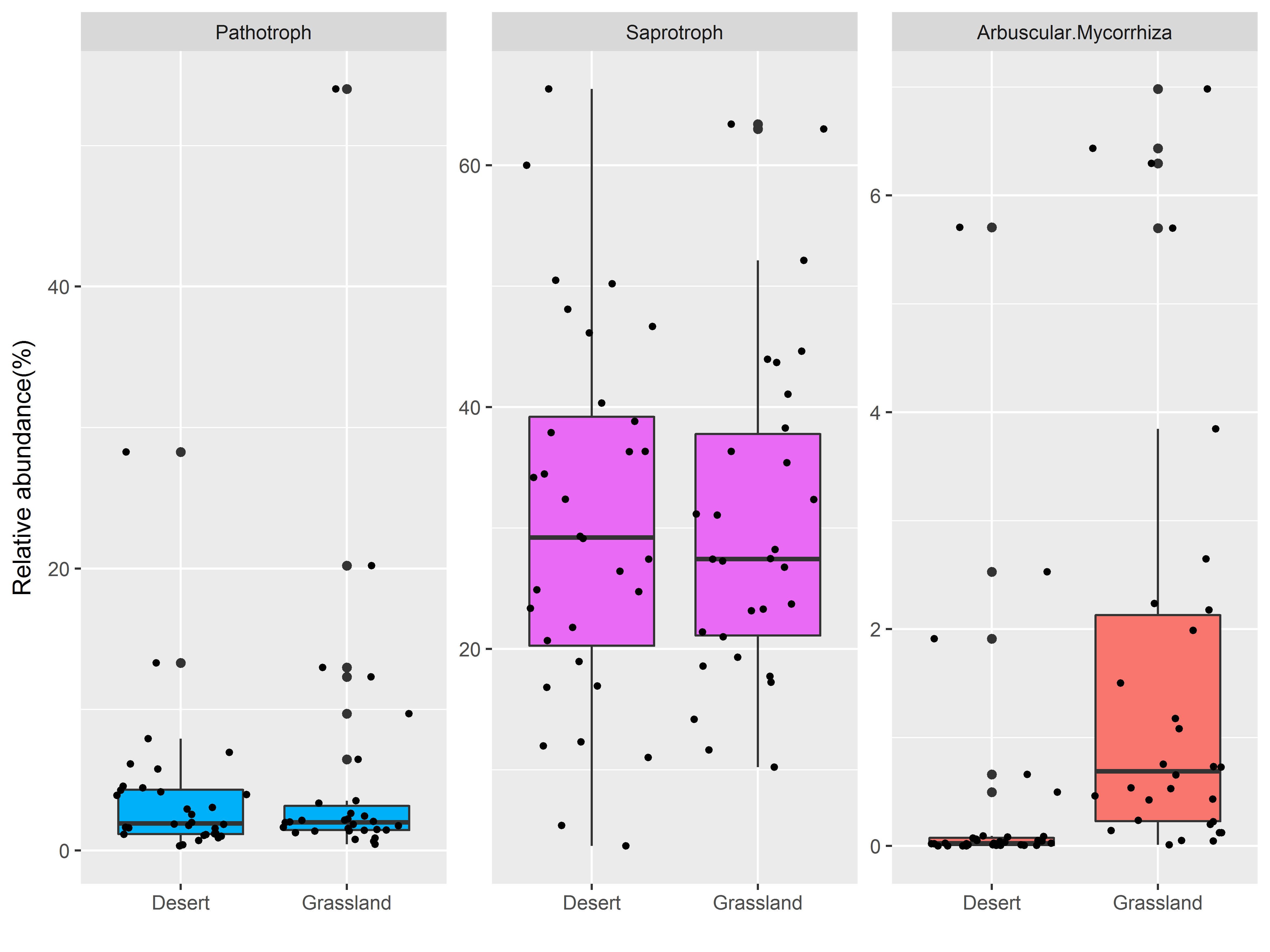

Supplement: Supplementary file 3 [file Image_3.TIF]

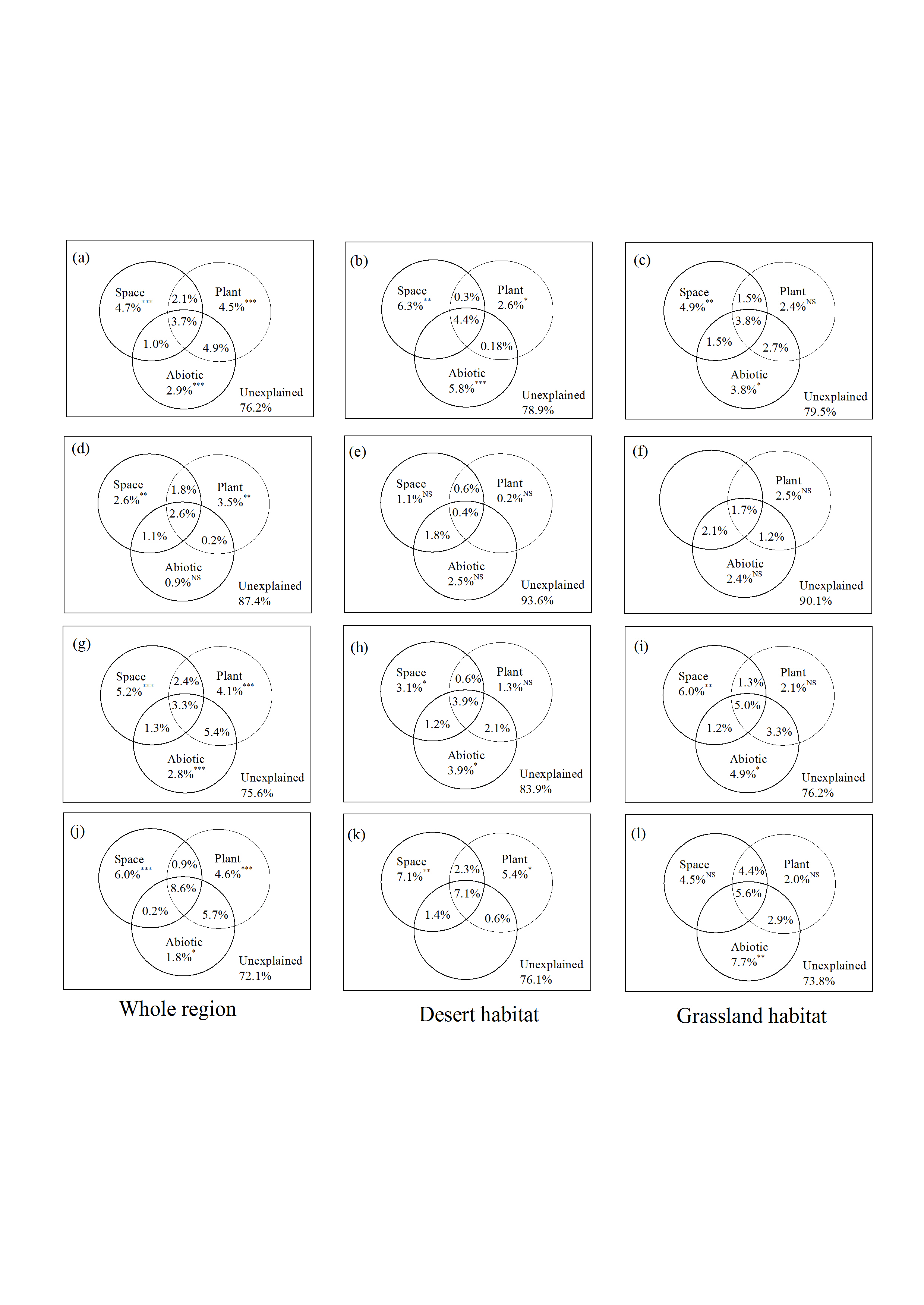

Supplement: Supplementary file 4 [file Image_4.JPEG]
