# Supplementary material for: Relationships Between Fungal and Plant Communities Differ Between Desert and Grassland in a Typical Dryland Region of Northwest China
Source: Front Microbiol. 2018 Oct 2;9:2327. doi: 10.3389/fmicb.2018.02327 (PMC6176009; doi:10.3389/fmicb.2018.02327)
Supplement: Supplementary file 5 [file Data_Sheet_1.DOC]

**Relationships between fungal and plant communities differ between** **desert and grassland in a typical dryland region of Northwest China**

Jianming Wang^1^, Chen Chen^1^, Ye Ziqi^1^, Jingwen Li**^1*^**, Yiming Feng^2^ and Qi Lu^2^

1 The College of Forestry, Beijing Forestry University, No. 35 Qinghua East Road, Haidian District, Beijing 100083, China.

2 Institute of Desertification Studies, CAF, Beijing, No.10 Huaishuju Road, Haidian District, Beijing, 100091, China.

E-mail: Lijingwenhy@bjfu.edu.cn

* Corresponding author

Telephone number: +8601062338100. E-mail address: Lijingwenhy@bjfu.edu.cn (J. L

Table S1 Summary statistics of the geographic and environmental characteristics for the desert, grassland and whole region

|  | Whole region (n = 62) | | Desert (n = 32) | | Grassland (n = 30) | |
| --- | --- | --- | --- | --- | --- | --- |
|  | Range | Mean | Range | Mean | Range | Mean |
| Longitude (°E) | 81.04~94.86 |  | 82.43~94.6 |  | 81.04~93.53 |  |
| Latitude (°N) | 41.93~47.56 |  | 41.93~47.56 |  | 43.45~47.13 |  |
| Altitude (m) | 216~2,153 | 1,217.27 ± 57.46 | 216~1,713 | **943.13 ± 62.71a** | 824~2,153 | **1,509.70 ± 64.49b** |
| MAP (mm) | 43~458 | 199.91 ± 11.42 | 43~226 | **154.41 ± 8.08a** | 94~458 | **248.43 ± 18.31b** |
| MAT (℃) | -0.60~9.00 | 4.15 ± 0.29 | 2.50~9.00 | **5.25 ± 0.32a** | -0.60~6.40 | **2.97 ± 0.40b** |
| Plant species richness | 6~19 | 11.13 ± 0.39 | 6~13 | **9.41 ± 0.41a** | 9~19 | **12.97 ± 0.51b** |
| Plant Coverage | 3.8~70.2 | 27.58 ± 2.11 | 3.8~32.1 | **15.43 ± 1.52a** | 22.50~70.20 | **40.55 ± 2.32b** |
| SM (%) | 0.02~32.29 | 4.95 ± 0.87 | 0.02~20.17 | **1.53 ± 0.63a** | 0.20~32.29 | **8.60 ± 1.39b** |
| TSN(g/kg) | 0.08~6.14 | 1.40 ± 0.18 | 0.08~2.11 | **0.53 ± 0.07a** | 0.44~6.14 | **2.33 ± 0.29b** |
| TSP(g/kg) | 0.26~1.14 | 0.58 ± 0.02 | 0.26~1.14 | 0.56 ± 0.04a | 0.31~0.96 | 0.59 ± 0.03a |
| TOC(g/kg) | 0.7~66.05 | 13.70 ± 1.90 | 0.70~11.85 | **4.73 ± 0.54a** | 2.78 -66.05 | **23.28 ± 3.05b** |
| AN(mg/kg) | 1.39~116.78 | 27.13 ± 3.02 | 1.39~116.78 | **19.53 ± 3.60a** | 9.80~105.48 | **35.25 ± 4.52b** |
| CN | 1.25~34.17 | 10.39 ± 0.61 | 1.25~34.17 | 10.88 ± 1.14a | 5.29~16.12 | 9.87 ± 0.37a |
| NP | 0.26~7.95 | 2.39 ± 0.28 | 0.26~4.44 | **1.03 ± 0.16a** | 0.90~7.95 | **3.83 ± 0.40b** |
| pH | 6.42~9.54 | 8.17 ± 0.08 | 6.76~9.54 | **8.42 ± 0.10a** | 6.42~9.22 | **7.90 ± 0.11b** |

MAP: mean annual precipitation; MAT: mean annual temperature; SM: soil moisture content; TSN: soil total nitrogen; TSP: soil total phosphorus; TOC: soil total organic carbon; AN: Soil available nitrogen; CN: soil carbon: nitrogen ratios; NP: soil nitrogen: Phosphorus ratios.

Values in bold represent the significant group difference in environmental variables between desert and grassland.

Table S2 Numbers of reads and OTUs belonging to the indicated functional groups

| Habitat | Functional groups | Total Reads | Mean Reads | Total OTUS | Mean OTUS |
| --- | --- | --- | --- | --- | --- |
|  | Total fungi | 1,211,922 | 19,547.13 ± 5.22 | 5,584 | 591.77 ± 18.61 |
|  | Arbuscular mycorrhizae | 11, 835 | 190.88 ± 40.80 | 189 | 16.39 ± 2.09 |
|  | Saprotroph | 371,098 | 5,985.45 ± 358.01 | 1,010 | 147.07 ± 4.47 |
|  | Pathotroph | 55,011 | 887.27 ± 198.71 | 179 | 25.81 ± 0.89 |
|  |  |  |  |  |  |
| Desert | Total fungi | 625,688 | 19,552.75 ±7.27 | 3,755 | 530.22 ± 21.03 |
|  | Arbuscular mycorrhizae | 2,364 | 73.87 ± 38.54 | 89 | 7.46 ± 1.51 |
|  | Saprotroph | 192,311 | 6,009.72 ± 530.37 | 785 | 137.38 ± 5.66 |
|  | Pathotroph | 24,129 | 754.03 ± 18.048 | 133 | 24.38 ±1.32 |
|  |  |  |  |  |  |
| Grassland | Total fungi | 586,234 | 19,541.13 ± 7.46 | 4,617 | 657.43 ± 26.71 |
|  | Arbuscular mycorrhizae | 9,471 | 315.70 ± 74.86 | 184 | 26.90 ± 3.05 |
|  | Saprotroph | 178,787 | 5,959.57 ± 486.21 | 834 | 157.40 ± 6.58 |
|  | Pathotroph | 30,882 | 1,029.40 ± 364.91 | 162 | 27.33 ± 1.16 |

Table S3 Summary statistics of the relationships between plant species richness and major functional fungal richness, and between plant community dissimilarity and major functional fungal community dissimilarity

| Habitat | Trophic groups | Plant Richness | | Plant community dissimilarity | |
| --- | --- | --- | --- | --- | --- |
|  |  | *R^2^*_adj_ | *P* | *R* | *P* |
| Whole | Arbuscular mycorrhiza | 0.196 | < 0.001 | 0.141 | < 0.01 |
|  | Saprotroph | 0.142 | < 0.01 | 0.356 | < 0.0001 |
|  | Pathotroph | 0.137 | < 0.01 | 0.442 | < 0.0001 |
|  |  |  |  |  |  |
| Desert | Arbuscular mycorrhiza |  | > 0.05 |  | > 0.05 |
|  | Saprotroph | 0.184 | < 0.01 | 0.155 | < 0.05 |
|  | Pathotroph | 0.210 | < 0.01 | 0.328 | < 0.0001 |
|  |  |  |  |  |  |
| Grassland | Arbuscular mycorrhiza |  | > 0.05 | 0.144 | < 0.05 |
|  | Saprotroph |  | > 0.05 | 0.326 | < 0.0001 |
|  | Pathotroph |  | > 0.05 | 0.308 | < 0.0001 |

Table S4 Relationships of the species richness of total, AM, saprotrophic, pathotrophic fungi and environmental variables

|  | Total fungi | Arbuscular mycorrhiza | Saprotroph | Pathotroph |
| --- | --- | --- | --- | --- |
| **Whole region** |  |  |  |  |
| MAP | 0.272(HS)*** | 0.157* | 0.301(HS)*** | 0.242(HS)*** |
| TSN | 0.251(HS)*** | 0.230*** |  |  |
| TOC | 0.245(HS)*** | 0.184*** |  |  |
| Plant Coverage | 0.228*** | 0.227*** |  |  |
| SM | 0.116** | 0.097** |  |  |
| NP | 0.09** |  |  |  |
| CN | 0.087* |  | 0.105** | 0.173*** |
| TSP |  |  | 0.143(HS)*** | |
| AN |  |  |  |  |
| pH |  |  |  |  |
| MAT |  |  |  |  |
|  |  |  |  |  |
| **Desert** |  |  |  |  |
| MAP | 0.097* |  |  | 0.151* |
| CN |  |  | 0.131* | 0.224** |
| Plant Coverage |  |  |  |  |
| SM |  |  |  |  |
| TSN |  | 0.129* |  |  |
| TSP |  |  |  |  |
| TOC |  | 0.115* |  |  |
| AN |  |  |  |  |
| NP |  |  |  |  |
| pH |  |  |  |  |
| MAT |  |  |  |  |
| **Grassland** |  |  |  |  |
| MAP | 0.262** |  | 0.286** | 0.116* |
| CN | 0.16* | 0.107* | 0.117* |  |
| AN | 0.107* |  |  |  |
| Plant Coverage |  |  |  |  |
| SM |  |  |  |  |
| TSN |  |  |  |  |
| TSP |  |  |  |  |
| TOC |  |  |  |  |
| NP |  |  |  |  |
| pH |  |  |  |  |
| MAT |  |  |  |  |

MAP: mean annual precipitation; MAT: mean annual temperature; SM: soil moisture content; TSN: soil total nitrogen; TSP: soil total phosphorus; TOC: soil total organic carbon; AN: soil available nitrogen; CN: soil carbon: nitrogen ratios; NP: soil nitrogen: Phosphorus ratios. *R*^2^ was reported if its significance level is < 0.05. HS represent the quadratic relatiohip between richness and variables. (***, *P* < 0.001;**, *P* < 0.01; *, *P* < 0.05).

Table S5 Environmental and spatial variables that significant correlated with total and major functional fungal community structure in desert, grassland and whole region are listed below. The correlation (*R*) and significance (*) were determined by Mantel tests based on 10000 permutations.

|  | Total fungi | Arbuscular mycorrhiza | Saprotroph | Pathotroph |
| --- | --- | --- | --- | --- |
| **Whole** |  |  |  |  |
| MAP | 0.581*** |  | 0.491*** | 0.482*** |
| MAT | 0.435*** | 0.123 | 0.408*** | 0.344** |
| Plant Coverage | 0.439*** | 0.122 | 0.389*** | 0.375*** |
| Geographic distance | 0.421*** | 0.204*** | 0.376*** | 0.309*** |
| TSN | 0.394*** |  | 0.318*** | 0.489*** |
| TOC | 0.378*** |  | 0.309*** | 0.521*** |
| NP | 0.373*** |  | 0.304*** | 0.465*** |
| pH | 0.354*** |  | 0.320*** | 0.218** |
| SM | 0.303** |  | 0.228* | 0.432*** |
| CN | 0.287** | 0.182* | 0.279** |  |
| AN | 0.256* |  | 0.188* | 0.351*** |
| TSP | 0.163* |  | 0.141 |  |
|  |  |  |  |  |
| **Desert** |  |  |  |  |
| Geographic distance | 0.521*** |  | 0.379*** | 0.318*** |
| CN | 0.467** |  | 0.391** | 0.2 |
| MAP | 0.458*** |  | 0.395*** | 0.2 |
| MAT | 0.378*** |  | 0.353*** | 0.2 |
| TSN | 0.309* |  | 0.262* |  |
| pH | 0.293 |  | 0.203 |  |
| AN |  |  |  |  |
| NP |  |  |  |  |
| SM |  |  |  |  |
| TSP |  |  |  |  |
| TOC |  |  |  |  |
| Plant Coverage |  |  |  |  |
|  |  |  |  |  |
| **Grassland** |  |  |  |  |
| MAP | 0.531*** | 0.345*** | 0.508*** | 0.348** |
| TOC | 0.404*** | 0.184 | 0.389*** | 0.298** |
| TSN | 0.363*** | 0.161 | 0.34* | 0.267* |
| pH | 0.367** |  | 0.333* | 0.202 |
| MAT | 0.345*** | 0.278** | 0.318** | 0.259*** |
| Plant Coverage | 0.333** | 0.175 | 0.296* | 0.189 |
| AN | 0.325** |  | 0.278* | 0.307** |
| SM | 0.300* | 0.208 | 0.21 | 0.256* |
| Geographic distance | 0.281* | 0.209 | 0.321* | 0.283*** |
| NP | 0.254** | 0.122 | 0.264*** | 0.189* |
| TSP | 0.256 |  | 0.183 |  |
| CN |  |  |  |  |

MAP: mean annual precipitation; MAT: mean annual temperature; SM: soil moisture content; TSN: soil total nitrogen; TSP: soil total phosphorus; TOC: soil total organic carbon; AN: Soil available nitrogen; CN: soil carbon: nitrogen ratios; NP: soil nitrogen: Phosphorus ratios. *R* was reported if its significance level is < 0.05. (***, *P* < 0.0001;**, *P* < 0.001; *, *P* < 0.01)

**Figure legends:**

**Figure S1.** Relative abundance (a) and numbers of fungal operational taxonomic units (OTUs) (b) belonging to major functional (trophic) groups.

**Figure S2.** Variation of relative abundance of the dominant fungal genera between desert and grassland samples.

**Figure S3.** Variation of relative abundance of the major functional fungi between desert and grassland.

**Figure S4.** Variation partitioning for the influence of plant, spatial and abiotic factors on total (a-c), AM (d-f), saprotrophic (g-i), pathogenic (j-l) fungal community composition in the desert (b, c, h, k), grassland (c, f, I, l) and whole region (a, d, g, j). (**, *P* < 0.01; *, *P* < 0.05; NS, *P* > 0.05)
